# Transcriptomic comparison reveals genetic variation potentially underlying seed developmental evolution of soybeans

**DOI:** 10.1093/jxb/ery291

**Published:** 2018-08-03

**Authors:** Huihui Gao, Yan Wang, Wei Li, Yongzhe Gu, Yongcai Lai, Yingdong Bi, Chaoying He

**Affiliations:** 1 State Key Laboratory of Systematic and Evolutionary Botany, Institute of Botany, Chinese Academy of Sciences, Xiangshan, Beijing, China; 2 University of Chinese Academy of Sciences, Beijing, China; 3 Crop Tillage and Cultivation Institute, Heilongjiang Academy of Agricultural Sciences, Harbin, Heilongjiang, China

**Keywords:** Differentially expressed gene (DEG), domestication, fruit development, RNA-seq, seed size, soybean

## Abstract

Soybean (*Glycine max*) was domesticated from its wild relative *Glycine soja*. However, the genetic variations underlying soybean domestication are not well known. Comparative transcriptomics revealed that a small portion of the orthologous genes might have been fast evolving. In contrast, three gene expression clusters were identified as divergent by their expression patterns, which occupied 37.44% of the total genes, hinting at an essential role for gene expression alteration in soybean domestication. Moreover, the most divergent stage in gene expression between wild and cultivated soybeans occurred during seed development around the cotyledon stage (15 d after fertilization, G15). A module in which the co-expressed genes were significantly down-regulated at G15 of wild soybeans was identified. The divergent clusters and modules included substantial differentially expressed genes (DEGs) between wild and cultivated soybeans related to cell division, storage compound accumulation, hormone response, and seed maturation processes. Chromosomal-linked DEGs, quantitative trait loci controlling seed weight and oil content, and selection sweeps revealed candidate DEGs at G15 in the fruit-related divergence of *G. max* and *G. soja*. Our work establishes a transcriptomic selection mechanism for altering gene expression during soybean domestication, thus shedding light on the molecular networks underlying soybean seed development and breeding strategy.

## Teaser

This work suggests the role of gene expression alteration at the transcriptomic level during soybean domestication, reveals genes which may underlie soybean seed development, and identifies genetic material that might potentially be used for soybean improvement.

## Introduction

Soybean [*Glycine max* (L.) Merr.], the most important crop among legumes, providing ~70% of dietary proteins and ~30% of edible oil worldwide (Lam *et al.*, 2010), was domesticated from its annual wild relative, *Glycine soja*, in China 5000 years ago (Hymowitz, 1970; Carter *et al.*, 2004). However, the genetic variations underlying soybean domestication are not well known. Genetic tools and resources have been developed for soybeans, but the progress of forward and reverse genetics has generally been slow. The reasons for this limited progress are mainly the genomic complexity and the difficulty of genetic transformation. A few genes have been found to be involved in soybean domestication and improvement through various strategies, such as mapping of quantitative trait loci (QTLs). These genes include *early maturity 1* (*E1*), *GIGANTEA* (*GI*), and the *juvenile* (*J*) locus, which regulate maturity and flowering time (Watanabe *et al.*, 2011; Xia *et al.*, 2012; Wang *et al.*, 2016; Lu *et al.*, 2017); *GmHs1-1*, which controls hard seededness (Sun *et al.*, 2015); *pod dehiscence 1*(*PDH1*), which confers pod-shattering resistance (Funatsuki *et al.*, 2014); and determinate stem locus (*Dt1* and *Dt2*), which controls determinate growth (Liu *et al.*, 2010; Ping *et al.*, 2014). However, increasing advances in sequencing technology have provided a genomic platform (Metzker, 2010) to predict the domesticated genes in various crops, such as rice (Huang *et al.*, 2012; Wang *et al.*, 2018), maize (Swanson-Wagner *et al.*, 2012), and tomato (Koenig *et al.*, 2013). This is having a significant impact on molecular breeding programs (Poland and Rife, 2012).

The soybean genome has 20 pairs of chromosomes. The first draft of the soybean genome (cultivar Williams 82) was released in 2010 and predicted the genome size to be 1115 Mb (Findley *et al.*, 2010; Schmutz *et al.*, 2010). Using the sequence draft as a reference, re-sequencing analyses of soybean populations and comparative genomics have greatly enhanced our ability to identify the genetic characteristics that distinguish cultivated and wild soybeans, which helps us to understand the genetic basis of cultivar phenotypic specializations (M.Y. Kim et al., 2010, 2012; Lam *et al.*, 2010; Chan *et al.*, 2012; Chung *et al.*, 2014; Li *et al.*, 2014; Zhou *et al.*, 2015; Fang *et al.*, 2017). Genome comparisons between wild and cultivated soybeans have revealed that only ~0.31% of the nucleotide sequences differ between them (M.Y. Kim *et al.*, 2010, 2012). The challenge now is to understand the functional consequences of the small fraction of these genetic changes and how they are involved in making cultivated soybeans different from wild soybeans. Much effort has focused on identifying genes that underwent large sequence changes or accelerated rates of nucleotide change in cultivated soybean lines (Chung *et al.*, 2014; Li *et al.*, 2014; Zhou *et al.*, 2015). This has led to the discovery of some genomic regions affected by artificial selection. These genomic regions are associated with agricultural traits, for example plant height, pubescence form, twinning trait, maximum internode length, number of nodes, and seed-oil content (Lam *et al.*, 2010; Zhou *et al.*, 2015). Although the genomic regions identified in previous studies are valuable for marker-assisted breeding (Lam *et al.*, 2010; Chung *et al.*, 2014), the high level of linkage disequilibrium in soybeans hinders the determination of causal genes in these regions related to soybean domestication (Lam *et al.*, 2010; Chan *et al.*, 2012; Chung *et al.*, 2014). A soybean pan-genome analysis has also identified genes, such as *senescence-associated gene 101* (*SAG101*), with amino acid changes resulting from large-effect single nucleotide polymorphisms (SNPs) and/or indels (Li *et al.*, 2014). These results indicate that changes affecting protein sequences are a source of genetic variation in soybean domestication.

Transcriptomics has made it possible to explore another dimension of domestication, namely changes in patterns of gene expression. Evidence for changes at the transcriptional level during domestication has also been examined in various crops. For example, a study in maize has suggested the widespread alteration of transcriptional networks during domestication (Swanson-Wagner *et al.*, 2012), which is consistent with suggestions that the regulation of gene expression has played an important role in the evolution of maize (Doebley and Lukens, 1998; Doebley *et al.*, 2006; Zhao *et al.*, 2008). The same has been found in tomato domestication (Koenig *et al.*, 2013). A transcriptome analysis was performed during early and middle seed maturation stages between wild and cultivated soybean varieties (Lu *et al.*, 2016), and it identified 2680 differentially expressed genes (DEGs) in these maturation stages. The genes encoding gibberellin 20 oxidase (*GA20OX*) and nuclear factor Y subunit A (*NFYA*) were found to increase seed size and seed-oil content, respectively, in transgenic Arabidopsis (Lu *et al.*, 2016). Another transcriptomic comparison of early maturation stages of developing seeds was performed between a cultivated soybean and a landrace with contrasting seed size phenotypes (Du *et al.*, 2017); both accessions are from the Yangtze River region, one possible origin of soybean domestication. A cytochrome P450 family gene (*GmCYP78A5*) was found to be differentially expressed in the two soybeans, and transgenic soybean lines overexpressing this gene exhibit enlarged seed size and increased seed weight (Du *et al.*, 2017). These transcriptome analyses provided the first sets of expression data on genes controlling the mid to mature stage of seed development, but they are not sufficient to enable a comprehensive understanding of the transcriptomic variation underlying seed developmental evolution, since domestic soybeans are believed to have multiple origins (Xu *et al.*, 2002).

To gain further insight into the developmental evolution of soybean fruit and seed, in the present study, we chose cultivated and wild soybeans from a major soybean-producing center, northeast China, which is also a predicted soybean domestication center (Fukuda *et al.*, 1933), and performed a transcriptome-wide analysis (see Supplementary Fig. S1 at *JXB* online). Analyses of the obtained 40 RNA sequencing (RNA-seq) data sets suggested that gene expression alteration was extensive between wild and cultivated soybeans, and analyses of gene expression clusters and DEGs suggested that most divergence in gene expression between wild and cultivated soybeans occurs around the cotyledon stage at the late pod development stage (G15, defined as 15 d pods after fertilization). Moreover, weighted gene network analysis identified one molecular module negatively associated with fruit and seed development at G15 in wild soybean, and the genes in this module, which are related to cell division, were expressed at low levels in wild soybean fruit relative to cultivated soybeans. Genome-wide linkage of DEGs, QTLs (seed weight and oil), and selection sweeps on chromosomes suggested that 157 DEGs at G15 between cultivated and wild soybeans were from domestication sweep regions, and 1528 and 1293 DEGs were associated with the identified QTLs controlling seed weight and oil content, respectively. Our work thus establishes that gene expression changes might have been preferentially targeted for fruit-related trait formation during soybean domestication. Our results provide new insights into soybean domestication and offer candidate genes to be considered during soybean improvement.

## Materials and methods

### Plant growth and sample collection

Seeds of wild soybean (*G. soja*) and cultivars (*G. max*) were collected from the northeast of China. Four accessions from each soybean species (wild and cultivated), as four repeats for each species, were chosen based on their rich diversity in color of flower and seed coat, seed size, and the content of protein and oil in seeds (Supplementary Dataset S1). They were grown in an experimental filed under natural conditions in May 2014 (Institute of Botany, Beijing, China). Apical buds (JJ) of 15-day-old seedlings after germination, flower buds (WH) and open flowers (H) of 1-month-old plants, and the developing pods at 5 d (G5) and 15 d (G15) after fertilization were harvested, and then the collected samples were quickly frozen in liquid nitrogen and stored at –70 °C for RNA isolation.

### RNA extraction, library construction, and RNA-seq

RNA was extracted using an SV Total RNA Isolation System (Promega, Madison, WI, USA) according to the manufacturer’s instructions. Forty libraries from the collected tissues or organs of wild and cultivated soybeans were constructed and sequenced using an Illumina HiSeq™4000, which was commercially performed by the Beijing Genomics Institute (BGI, http://www.genomics.cn/index).

### Processing of reads

Clean reads were obtained through the following three steps: (i) reads with adaptor contamination were removed; (ii) reads with ambiguous sequences >5% were discarded; and (iii) low quality reads that contained a >20% quality score (Q-score) <20 bases were removed. Trimmed RNA-seq reads were mapped to the reference genome using Bowtie v2.1.0 (Langmead and Salzberg, 2012) and TopHat 2.0.9 (Kim *et al.*, 2013). The *G. max* reference genome of Williams 82 (version a2.v1) was downloaded from Phytozome10 (Goodstein *et al.*, 2012).

### Gene expression quantification

The RSEM v1.2.12 package was used to calculate gene expression levels for each sample and normalized by fragments per kilobase of transcript per million mapped reads (FPKM) (Trapnell *et al.*, 2010; Li and Dewey, 2011). If the FPKM was zero, we treated these genes as non-checked genes. The DEGs (fold change >2, Q-value >0.8) were identified using NOISeq (http://www.bioconductor.org) (Tarazona *et al.*, 2011).

### *De novo* assembly

Clean reads were assembled using the *de novo* assembly software Trinity v2.0.6 (Grabherr *et al.*, 2011). First, clean reads with a certain length of overlap were combined to generate contigs. Then, the paired-end reads were realigned to contigs to obtain unigenes, which could identify different contigs in the same transcript and ensure the interval among these contigs. The contigs in one transcript were assembled by Trinity and gained the sequences not extended on either end, which was defined as a unigene (Wang *et al.*, 2013).

### Functional annotation

Annotation analyses were performed by BLASTing publicly available protein databases, including Nr (http://www.ncbi.nlm.nih.gov), GO (http://www.geneontology.org), COG (http://www.ncbi.nlm.nih.gov/COG), SwissProt protein (http://www.expasy.ch/sprot), and KEGG (http://www.genome.jp/kegg). The best alignments were used to decide sequence direction and to predict coding regions of the unigenes. ESTScan (v3.0.2) software was used to decide sequence direction and coding regions when a unigene was aligned to none of the above databases (Iseli *et al.*, 1999). AgriGO was used to identify over-represented Gene Ontology (GO) terms (Du *et al.*, 2010). Functional categories were based on biological processes that were summarized using REViGO (Supek *et al.*, 2011) The unigenes were also aligned to the COG database to predict and classify possible functions. In addition, KEGG was used to annotate the pathways of the unigenes.

### Phylogenetic analyses and evolutionary analysis

A Neighbor–Joining (NJ) tree was built using transcriptome data from wild and cultivated soybean SNPs by MEGA6 (Tamura *et al.*, 2013). Protein-coding genes from four wild and four cultivated soybeans were used for gene family identification. Protein sequences for genes were compared by using all-by-all BLASTP (Altschul *et al.*, 1990), and then OrthoMCL v2 (Li *et al.*, 2003) was used to cluster genes into orthologous gene families. *De novo* contigs assembled from our reads, each unigene pair with >150 bp and 60% identity between orthologous gene pairs with one copy from each accession, were chosen as transcripts for single-copy genes. Once a unique gene was clearly mapped in Williams 82, it was defined as a conserved single-copy gene family in soybean. Protein sequences from the identified single-copy orthologous gene families were aligned by MUSCLE (v3.8.31) (Edgar, 2004). Four-fold degenerative sites from the coding sequence (CDS) alignments were extracted and concatenated for phylogenetic analysis, and the NJ method was incorporated in PhyML (v3.0) (Guindon *et al.*, 2010).

The Codeml program in the PAML package with a free ratio model (model=1) was used to estimate the evolutionary rate along each lineage for the eight accessions (Yang, 2007). The resulting Codeml data (such as the dN, dS, and dN/dS values) for the genes of each accession were calculated. Genes with dS=0 were filtered. To identify fast evolving genes (FEGs) and positively selected genes (PSGs), a branch model was used by setting the *G. max* branch as the foreground and *G. soja* as the background. Genes were considered to be FEGs if they had a higher value in the foreground branch than in the background branches. FEGs having dN/dS >1 were reported as PSGs.

### Gene clustering and visualization

K-means clustering was used to visualize genes exhibiting a similar expression pattern, and it was performed on log_2_-transformed FPKM values using MeV v4.8.1 (Saeed *et al.*, 2003) with Pearson’s correlation as similarity metrics.

### Weighted gene co-expression network analysis (WGCNA)

A gene co-expression network was constructed using the R package WGCNA (Langfelder and Horvath, 2008). The modules were obtained using the automatic network construction function blockwiseModules with default settings, with minor modifications (the power was 14, TOM-Type was adjacency, minModuleSize was 50, and mergeCutHeight was 0.25). The eigengene value was calculated for each module and used to test the association with each tissue type. The total connectivity, intramodular connectivity, and kME *P*-value were calculated. The hub genes in a given module were defined by kME >0.95, which measures a gene’s connectivity in the specific module. To characterize those modules, the GO enrichment analysis was tested by AgriGO with a *P*-value <0.05, and KEGG pathways with *P*-values <0.05 were considered to be significantly enriched.

### Visualization and plotting of genomic data

Gene distributions on chromosomes were visualized using MapChart2 (Voorrips, 2002). The QTL information was collected from Soybase (Grant *et al.*, 2010; http://soybase.org). The correlations between DEGs, QTLs, and selection sweeps were evaluated, and *P*<0.05 was considered to be significant.

### Statistical analyses

Statistical analyses were performed by using IBM SPSS Statistics for Windows, Version 24.0 (IBM Corp, NY, USA).

### Data deposition

The RNA-seq data reported in the article have been deposited in the database of the National Center for Biotechnology Information (NCBI) under accession number SRP154393 (https://www.ncbi.nlm.nih.gov/sra/SRP154393).

## Results

### Qualitative evaluation of RNA-seq data

To observe general variation patterns at the transcriptomic level between cultivated and wild soybeans, four accessions of each species that showed tremendous phenotypic variations were chosen from the northeast region of China as representatives, and were also used as four independent repeats. For convenience, the four wild accessions were abbreviated to Y1, Y2, Y3, and Y4, while the four cultivated soybeans were abbreviated to DN, HF, HN, and NF (for details, see Supplementary Dataset S1). They displayed a rich diversity in flower color, seed coat color, seed size, oil content, protein content, and isoflavone content (Supplementary Dataset S1), suggesting full representation of wild and cultivated soybeans in this geographic region. For comprehensive evaluation, apical buds (JJ), flower buds (WH), flowers (H), and the developing pods at 5 d (heart stage) and 15 d (cotyledon stage) after fertilization (respectively referred to as G5 and G15) were collected from each accession of the two soybean species and subjected to RNA-seq analysis.

Each sample was named with the abbreviated accession name plus the tissue name in certain cases, and 40 RNA-seq libraries were constructed and sequenced in total (Supplementary Dataset S2). After adaptor trimming, an average of 59 million clean reads per library was acquired after RNA-seq (Supplementary Dataset S2). The genomic sequences of the selected accessions are unknown; however, previous studies suggest that only ~0.31% nucleotide difference is found between *G. max* and *G. soja* (M.Y. Kim *et al.*, 2010, 2012). Therefore, the genome of Williams 82 (a2.v1) (Schmutz *et al.*, 2010) was used as a reference in our analysis. The clean RNA-seq reads were mapped onto the genome reference, generating an average of 85.66% and 83.88% gene map rates, respectively, for the cultivated and wild soybean (Supplementary Dataset S2), thus providing a fair basis for comparison. Referenced by the Williams 82 genome, 35274 and 33533 genes were detected, respectively, in all investigated tissues of cultivated and wild soybeans, and 32829 were simultaneously expressed in all included tissues (Supplementary Dataset S3). *Glycine soja* had 2445 genes that were not expressed in at least in one, while *G. max* had 704 such genes, among which only one gene (Glyma.08G198300) that encodes glucose-6-phosphate 1-dehydrogenase was not detected in any examined tissues of *G. max* (Supplementary Dataset S3). Altogether, we detected 52255 soybean genes in the involved soybean tissues.

### Sequence diversity among soybeans

We first evaluated the sequence diversity of soybeans using the obtained transcriptomic data. By alignment to the Williams 82 genomic sequence, SNP sites in coding and non-coding regions including untranslated regions (UTRs) and introns of each gene in each tissue pair were analyzed between wild and cultivated accessions (Supplementary Dataset S4). More SNPs seemed to exist in each region of transcripts from wild soybeans, but the differences were not significant between most tissue comparisons compared with cultivated soybeans. However, the analysis revealed a total of 71318 ± 15145 SNPs among the four cultivars and 98131 ± 29025 among the four wild accessions, and this was significantly different between *G. soja* and *G. max* (Supplementary Fig. S2A, B; Supplementary Dataset S4), suggesting an overall higher level of transcript diversity in wild soybeans than in cultivated ones.

**Fig. 1.**
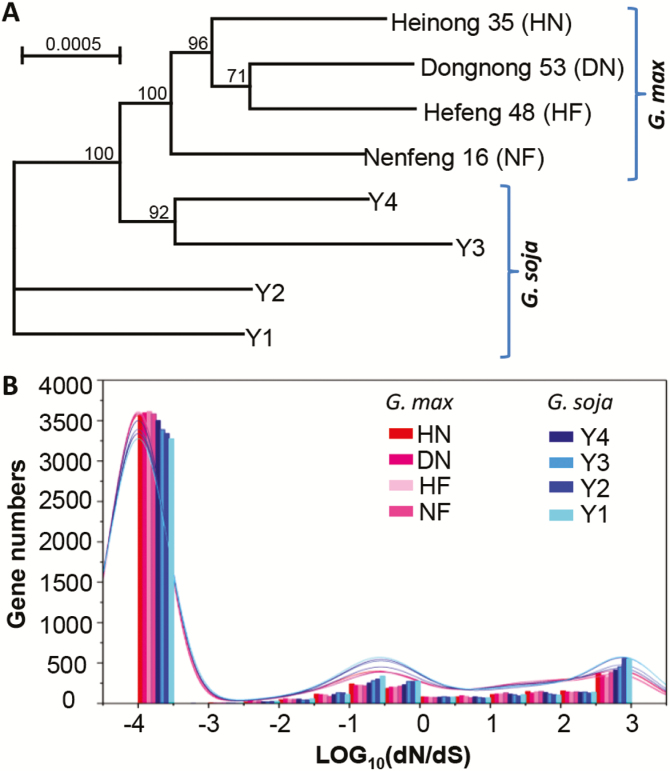
Sequence diversity of wild and cultivated soybeans. (A) Unrooted NJ tree of wild and cultivated soybeans using the identified orthologous genes. The scale bar represents the expected number of substitutions per site. (B) The distribution of the dN/dS ratio. The red, purple, pink, deep pink, dark blue, blue, gray, and turquoise lines represent dN/dS distributions of single-copy orthologous genes of cultivated soybeans (HN, DN, HF, and NF) and wild soybeans (Y4, Y3, Y2, and Y1).

Phylogenetic analysis based on the transcriptomic data revealed that the selected soybean cultivars formed a monophyly, while obvious separation was observed in the wild accessions (Supplementary Fig. S2C ). For rigorous analysis, we identified the strictly orthologous unigenes (single-copy genes) between the wild and cultivated soybeans. Altogether, 4987 orthologous unigene pairs were characterized (Supplementary Dataset S5). Phylogenetic analysis using these unigenes revealed a quite similar topology (Fig. 1A) to the tree based on transcriptomic data (Supplementary Fig. S2C), indicating a solid phylogeny for these soybeans.

If the ratio of non-synonymous (dN) to synonymous nucleotide substitutions (dS) is >1, this is indicative of positive selection, whereas dN/dS values equal to or significantly greater than 1 represent either purifying or neutral selection (Doron *et al*., 2005). We therefore evaluated the dN/dS ratios of the identified orthologous unigenes between the cultivated and wild soybeans. Based on the above-constructed phylogenetic tree (Fig. 1A), the dN/dS for each orthologous unigene pair was evaluated in the different branches using a free ratio model (model=1), which allows for a separate dN/dS ratio for each branch. We found that all four wild soybean branches (Y1, Y2, Y3, and Y4) had higher dN/dS ratios than the remaining branches of the four cultivated soybeans (DN, HF, HN, and NF) (Fig. 1B). Branch site analyses further revealed 8 and 14 FEGs, respectively, from the *G. max* branches and the *G. soja* branches, and 12 of them were functionally annotated (Supplementary Dataset S5 ). Among these FEGs, two PSGs from each soybean species were found and two were functionally annotated, which included one basic helix–loop–helix (bHLH) transcription factor (TF) gene (Glyma.13G368700) in *G. soja* and a WD repeat-containing protein gene (Glyma.06G185900) in *G. max*. These results indicated that only a small portion (0.44%) of the identified orthologous genes might have been rapidly evolving during soybean domestication.

### Divergence in gene expression patterns between wild and cultivated soybeans

**Fig. 2.**
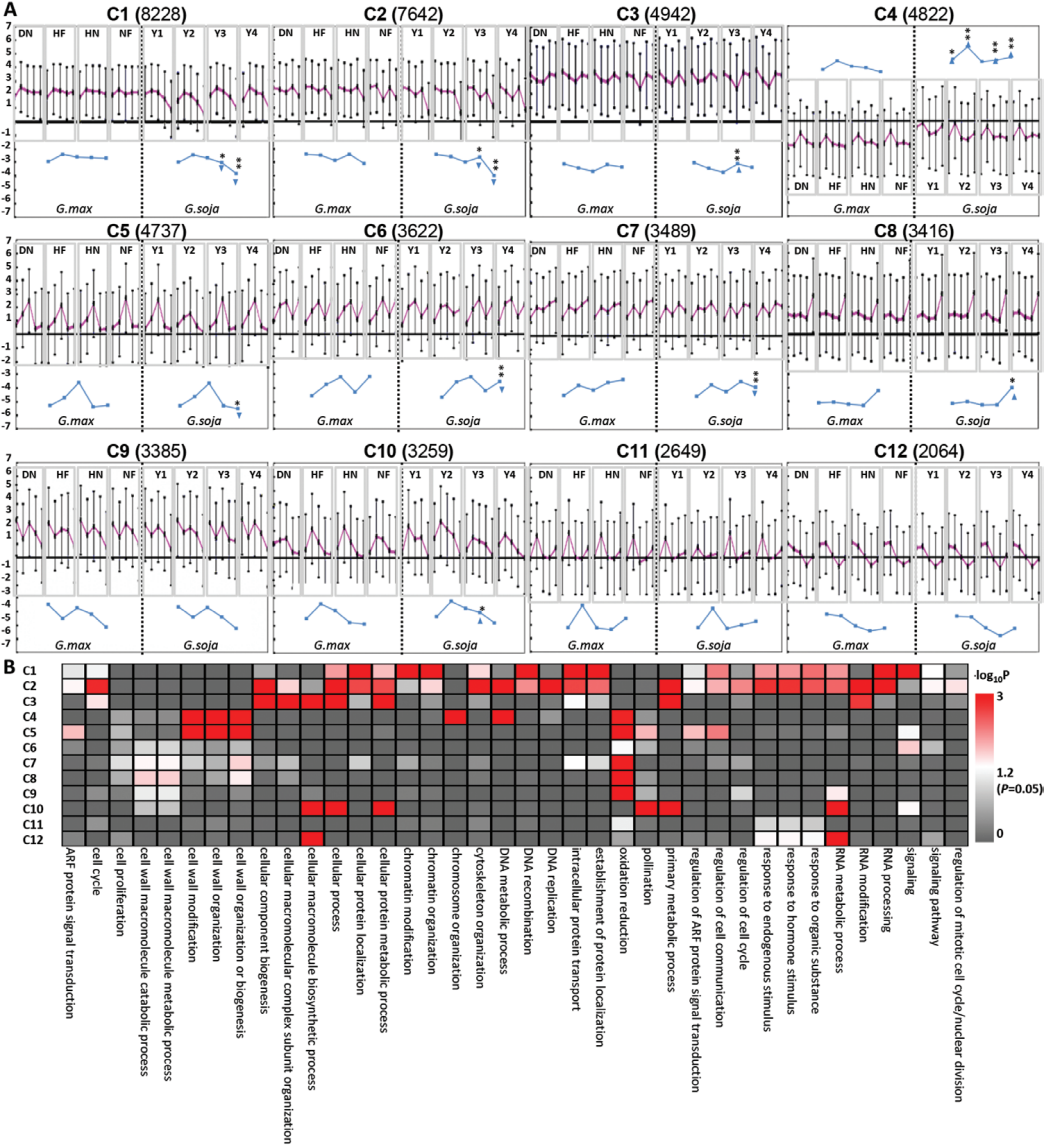
Gene expression pattern between wild and cultivated soybeans. (A) Gene clusters identified in 40 samples. Twelve gene clusters (C1–C12) were identified using k-means clustering. In each cluster, the *y*-axis represents log_2_ FPKM derived from RNA-seq data for each biological sample, while the *x*-axis represents the biological samples that are ordered as JJ, WH, H, G5, and G15 for each accession as indicated by DN, HF, HN, NF, Y1, Y2, Y3, and Y4, respectively. The blue curve represents the average expression of genes in each tissue of four accessions for both *G. max* and *G. soja*. Statistical differences (*P<0.05, **P<0.01) are indicated by Student’s *t*-test. The inverted triangles indicate down-regulation, and the upright triangles indicate up-regulation. (B) Heatmap of –log_10_*P* of biological process category enrichment by AgriGO among the 12 clusters.

To inspect the overall gene expression pattern between wild and cultivated soybeans, we used all transcriptomic data to perform K-means clustering analysis. This analysis allows measurement of the dynamic expression of genes during a time series comprising different tissues, including apical buds, floral buds, flowers, G5, and G15. The detected soybean genes (52255) were clustered into 12 gene co-expressed clusters designated as C1–C12, and the gene number for these clusters ranged from 2064 (C12) to 8228 (C1) (Fig. 2A; Supplementary Dataset S6). The genes in each cluster in principle show a similar expression pattern, while genes in different clusters feature distinct expression patterns. However, considering the gene expression levels in each cluster, significant differences in certain tissues were detected between wild and cultivated soybeans. Three clusters (C1, C2, and C4) were obviously divergent since at least two tissues with significant differences were detected between wild and cultivated soybeans, while the remaining nine clusters had overall similar and ‘common’ variation patterns, since occasionally at most one tissue showed differences between wild and cultivated soybeans in some clusters (Fig. 2A). Moreover, the C4 cluster was unique in showing extremely low gene expression in soybeans, and the differences seemed to be more significant (Fig. 2A). Interestingly, the significant differences detected in the clusters with ‘common’ variation patterns were detected at either G5 or G15 stages, while they were detected in both G5 and G15 among the three ‘divergent’ clusters (Fig. 2A), hinting at significant gene expression variation in fruit development between wild and cultivated soybeans. Nonetheless, the gene numbers in C1, C2, and C4 between wild and cultivated soybeans were ~37.44% of the total genes investigated.

To analyze further the functional significance of these genes, over-represented GO terms of the 12 clusters were identified, and each cluster apparently had distinctly enriched GO terms (Fig. 2B). A total of 32–229 significant terms were detected in the remaining 12 clusters (Supplementary Dataset S7). We particularly focused on the three divergent clusters C1, C2, and C4. Genes in C4 were enriched in GO terms of cell wall-related processes, DNA metabolic processes, and chromosome organization (Fig. 2B; Supplementary Dataset S7). Enrichment in C1 and C2 included genes involved in the accumulation of storage compounds and cell division processes (Fig. 2B; Supplementary Dataset S7). The genes in C1 were found to be mainly involved in β-glucan metabolic processes/metabolic processes, cellular macromolecule metabolic processes, cellular localization, protein modification/catabolism, chromatin modification/organization, and cell division processes (Fig. 2B; Supplementary Dataset S7). In particular, the genes involved in cell division processes included 11 involved in cell division, 29 related to cyclin, and 485 TF genes that encoded auxin response factors (ARFs), bHLH protein, WRKYGQK (WRKY)-containing protein, and some others (Supplementary Datasets S6, S7). The Arabidopsis homologs of some of these genes were found to function in cell division or affect seed size and maturation, for example the cell division control 48 (CDC48) gene and general transcription factor group E4 (GTE4)-like gene (Park *et al.*, 2008; Airoldi *et al.*, 2010). The genes in C2 were enriched in cell cycle (38 genes) and cell division (21 genes), including genes encoding cell cycle checkpoint-related protein RAD9 homolog A-like and cell division control protein CDC7-like (Fig. 2B; Supplementary Dataset S6). The orthologs of these genes in Arabidopsis drive the eukaryotic cell division cycle (Sabelli *et al.*, 2013). These genes in both C1 and C2 were significantly down-regulated, but in C4 they were significantly up-regulated in G5 and G15 of wild soybeans compared with cultivated soybeans (Fig. 2A), indicating that these genes may contribute to fruit development (i.e. seed size).

### A negatively correlated gene co-expression module in fruit development was found in wild soybeans

**Fig. 3.**
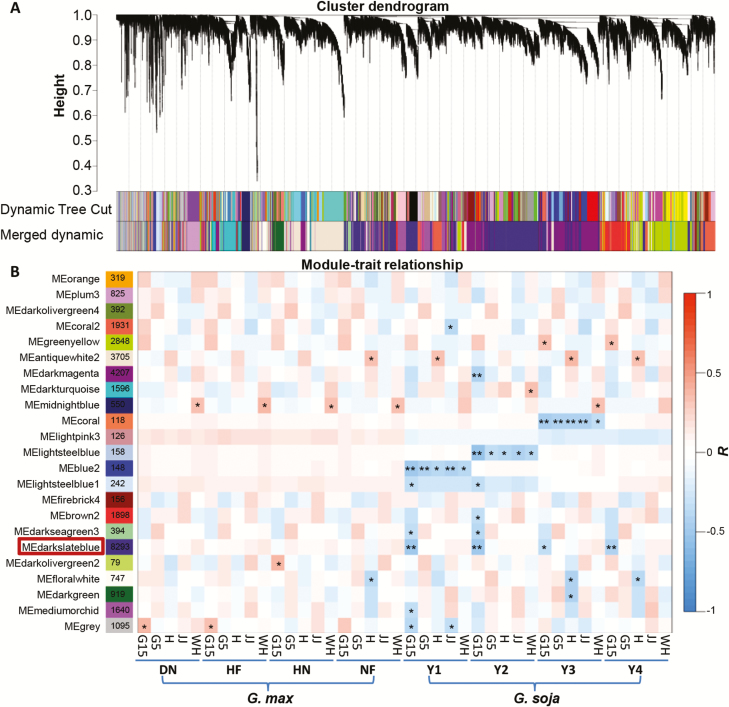
WGCNA based on the gene expression matrix from cultivated and wild soybeans. (A) Hierarchical cluster tree showing co-expression modules identified by WGCNA. Each leaf in the tree represents one gene. The major tree branches constitute 23 modules labeled with different colors. (B) Module–sample association. Each row corresponds to a module labeled with a color as in (A) Modules are distinguished by different colors which were arbitrarily assigned by the WGCNA package. Each column corresponds to a tissue type as indicated. The color of each cell at the row–column intersection indicates the correlation coefficient (*R*) between the module and the tissue type. *Significance at *P*<0.05; **Significance at *P*<0.01.

WGCNA is a powerful tool for identifying which sets of genes/functional pathways are linked to phenotypes. We therefore built weighted gene co-expression networks of soybeans using our RNA-seq data. In these analyses, co-expression networks were constructed based on pairwise correlations between genes in their common expression trends across all sampled tissues. Modules are defined as clusters of highly interconnected genes; genes within the same cluster have high correlation coefficients with one another. To reduce noise, genes with FPKM >5 in each tissue were included. According to this standard, a total of 32386 genes were subjected to WGCNA. The topology of the hierarchical clustering of samples clearly suggested the suitability of the selected genes for WGCNA (Supplementary Fig. S3A), while analysis of network topology for various soft-thresholding powers was set as 14 (Supplementary Fig. S3B). After these tests and settings, we ultimately discovered 23 distinct modules (labeled by different colors, Fig. 3A). The gene numbers in these modules ranged from 79 (darkolivergreen2) to 8293 (darkslateblue) (Fig. 3B; Supplementary Dataset S8). Notably, 17 out of 23 co-expression modules were comprised of genes that were significantly correlated with at least one single sample type (*P*<0.05; Fig. 3B). We observed three modules that negatively correlated with all five tissues: blue2, lightsteelblue, and coral from Y1, Y2, and Y3, respectively. Interestingly, we found that the darkslateblue module showed significant and negative correlation with the G15 tissues in all wild soybeans only (boxed in red, Fig. 3B), indicating putatively important roles for this module in soybean fruit and seed development.

**Fig. 4.**
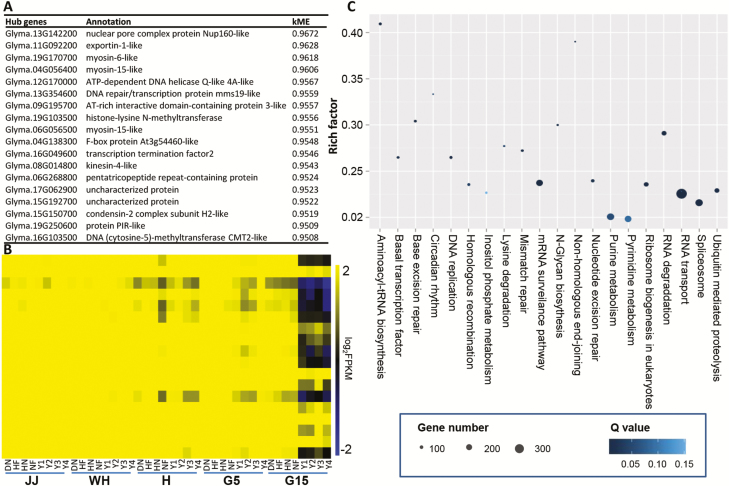
Characteristics of the darkslateblue module. (A) Annotation of hub genes (kME>0.95, *r*>0.85). (B) Heatmap of hub gene expression. The biological samples are arranged as indicated, and the genes are ordered in the same sequence as in (A). The gene expression is defined as log_2_ FPKM derived from RNA-seq data for each biological sample. (C) Top 20 statistics of KEGG pathway enrichment. KEGG pathways with *P*<0.05 are listed. Rich factor is the ratio of gene numbers to all gene numbers annotated in the KEGG pathway. The number of genes annotated in a specific KEGG pathway is expressed as the size of the circle. The *P*-value was corrected to a Q value ranging from 0 to 1.

The module eigengenes (also called hub genes) are those that show the most connections in the network as indicated by their high kME (eigengene connectivity) value. They are the first principal component of a given module and can be considered as representative of the module’s gene expression profile. Genes (kME >0.95) in each cluster were chosen as hub genes, and ultimately 287 eigengenes (ranging from 2 to 42 within the modules) were selected Supplementary Dataset S9). The module eigengenes for the 23 distinct modules were each correlated with distinct tissue types (samples) due to their tissue-specific expression profiles. Eighteen hub genes were identified in the darkslateblue module, and they were indeed expressed at a low level in G15 of wild soybeans compared with cultivated soybeans (Fig. 4A, B). Functional annotations suggested that 16 hub genes, which included three myosin-like genes, might be involved in multiple processes (Fig. 4A). The homologs of these genes in Arabidopsis are required for the cell cycle-regulated transport of various organelles and proteins for their segregation (Prokhnevsky *et al.*, 2008). Fifty-five genes in this module that are interconnected with these hub genes were also found to be associated with cell division, cell expansion, and thereby organ size (Supplementary Datasets S8, S9), including Glyma.13G049400 and Glyma.18G279500. Both genes are homologs of cell division protein 27B (AT2G20000) and 27A (AT3G16320) in Arabidopsis that respectively link the plant cell cycle to the progression of cell differentiation (Blilou *et al.*, 2002) and increase the growth rate and organ size (Rojas *et al.*, 2009). *GmGA20OX*, which enhances seed size/weight in soybeans (Lu *et al.*, 2016), was also in this module. Moreover, ~241 TF genes, such as WRKY, bHLH, APETALA2 (AP2), and GTE, were found in this module (Supplementary Dataset S8).

We further characterized the genes in the darkslateblue module. GO enrichment analysis revealed that the enriched GO terms in the darkslateblue module were indeed related to cell cycle, cell cycle checkpoint, microtubule-based movement, cellular processes, cellular protein localization, cellular macromolecule localization, and response to hormone stimulus (Supplementary Dataset S10), while KEGG enrichment analysis revealed that the darkslateblue module was enriched in DNA replication, *N*-glycan biosynthesis, RNA transport, and mismatch repair pathways (Fig. 4C).

Therefore, genes in the darkslateblue module were largely related to cell division and cell cycle processes, and expressed at low levels at G15 of wild soybeans relative to cultivated soybeans, which might lead to weak cellular activities such as cell division and cell expansion, thus resulting in small seed size in wild soybeans. Moreover, the expression of 65.22% genes in this module was lower in wild soybeans than in cultivated soybeans, implying potentially important roles for DEGs.

### Characterization of DEGs between wild and cultivated soybeans

**Fig. 5.**
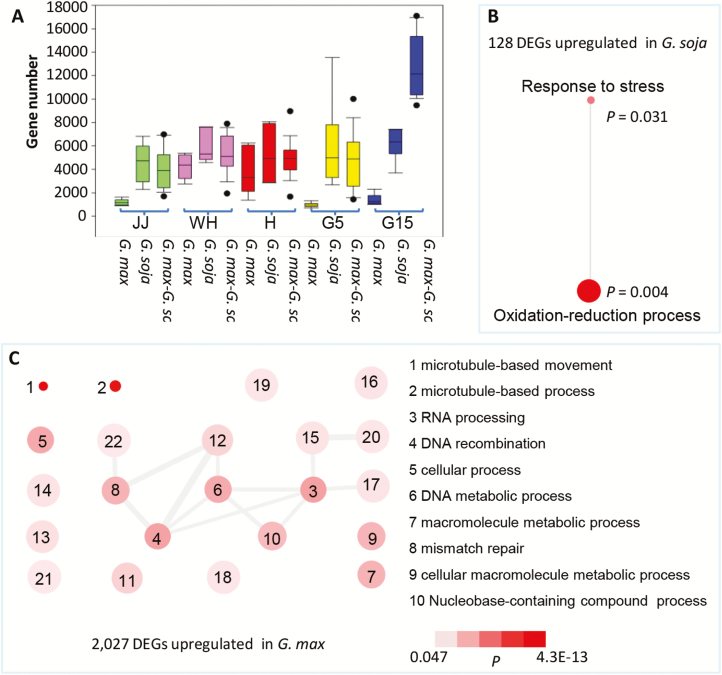
Characterization of DEGs between wild and cultivated soybeans. (A) DEG number variation in each tissue as indicated between cultivated and wild soybeans. (B) GO enrichment of genes that were up-regulated in wild soybeans. (C) GO enrichment of genes that were up-regulated in cultivated soybeans. For simplicity, the top 10 enriched GO terms are listed while the others are available in Supplementary Dataset S12. Functional categories were based on biological processes that were summarized using REViGO. The disc color indicates *P*-values for hypothesis testing as shown in the color bar, while disc size is proportional to log_2_ the number of genes in the category, indicating the frequency of the GO term in the underlying GOA database. Highly similar GO terms are linked by edges in the graph, where the line width indicates the degree of similarity.

We next evaluated the number of DEGs under a false discovery rate (FDR) ≤0.001 and log_2_ ratio ≥2 (cultivated/wild) in each tissue among the investigated soybeans, and we found that the number of DEGs varied from 30 to 2155 among the tissues, and altogether 2361 DEGs were found in soybeans (Supplementary Dataset S11), occupying 4.52% of total detected genes. Moreover, intraspecific variations in the number of DEGs among cultivated soybeans were always smaller than those among wild soybeans in the five tissues examined (Fig. 5A), again indicating that the wild soybeans were more divergent than the cultivated ones. Interestingly, the number of interspecific DEGs between wild and cultivated soybeans was comparable with that among wild soybeans in the developmental tissues of JJ, WH, H, and G5. However, the number of DEGs among wild soybeans and the interspecific DEGs clearly deviated from that among the cultivated soybeans at the G15 stage (Fig. 5A), indicating that gene expression at G15 was at its most divergent between wild and cultivated soybeans.

We thus focused on DEGs at G15, and ultimately identified 2155 DEGs in fruits (including seeds) between cultivated and wild soybeans at this developmental stage (Supplementary Dataset S11). Among these DEGs, 2027 genes were up-regulated and 128 genes were down-regulated in cultivated soybeans compared with wild soybeans. A total of 518 genes encoded unknown proteins, while 1637 genes encoded homologs of functionally annotated proteins. Notable genes down-regulated in cultivars but up-regulated in wild soybeans included six seed maturation protein genes (Glyma.03G144400, Glyma.13G363300, Glyma.19G147200, Glyma.17G155000, Glyma.20G147600, and Glyma.20G233000), and the accumulation of these proteins could be a critical event in seeds acquiring desiccation tolerance (Shih *et al.*, 2012). These genes may be related to a low seed biomass response to dehydration in wild soybeans. Many up-regulated genes in cultivated soybeans, including 61 TFs, including bHLH, ARFs, and MYB, are putatively related to cell division and cell expansion (Supplementary Dataset S11), since some homologs of these soybean genes in Arabidopsis can lead to extra cell division and expansion, thus increasing seed size and weight. For example, Glyma.11G204200 encodes the homolog of Arabidopsis ARF8, which is required to initiate fruit and seed development (Goetz *et al.*, 2006). Also, both Glyma.01G179800 and Glyma.02G059900 encode homologs of *DA1* (AT1G19270) in Arabidopsis that regulates organ and seed size (Li *et al.*, 2008).

We further performed GO enrichment analysis and found that the up-regulated genes in *G. soja* were exclusively enriched in two highly similar GO terms of response to stress and oxidation–reduction processes (Fig. 5B; Supplementary Dataset S12), while the significantly enriched GO terms in the up-regulated genes in *G. max* were related to multiple processes including microtubule-based movement/processes, RNA processing, DNA replication, cellular processes, and macromolecule metabolism (Fig. 5C; Supplementary Dataset S12). Moreover, DEGs involved in macromolecule metabolic processes included genes encoding lipoxygenase, sucrose synthase, and sugar transporters (Supplementary Dataset S11), which are related to the synthesis and accumulation of various storage compounds. These results further suggest putative roles for DEGs in soybean fruit and seed development.

Interestingly, 80.42% of DEGs at G15 fell into the three identified ‘divergent’ gene expression clusters, including 1217 and 502 DEGs at G15 in C1 and C2, respectively, while only 14 DEGs were in C4 (Supplementary Fig. S4A; Supplementary Dataset S11). Moreover, 83.43% of genes of the largest module (darkslateblue) came from C1 and C2 (Supplementary Dataset S8), while 69.79% of DEGs at G15 fell into the darkslateblue (1059 DEGs) and darkmagenta (445 DEGs) modules (Supplementary Fig. S4B; Supplementary Dataset S11). Furthermore, the proportion of DEGs in the hub genes of the two modules nearly occupied total DEGs in all module hub genes (Supplementary Fig. S4C). The DEGs, although fewer in most cases in other developmental tissues, in addition to ‘common’ gene expression clusters and other co-expression modules (Supplementary Fig. S4), need further analysis; however, DEGs at G15 might have been essential players during soybean domestication.

### Chromosomal distribution of DEGs in domestication sweeps

Analysis of the relationship between gene transcription and chromosomal location aids in our understanding of the regional dynamics of chromosome evolution. To create a soybean transcriptome map and globally observe DEGs between cultivated and wild soybeans, we next investigated the physical distribution of DEGs at G15 on 20 soybean chromosomes (Supplementary Fig. S5). The gene expression levels were estimated by the average values of four accessions of each species. The correlation between the DEG number and gene density was calculated on each chromosome. In line with a previous study (Libault *et al.*, 2010), we found that the expression of genes in pericentromeric regions was lower than that in chromosomal arms (Supplementary Fig. S5). Moreover, the expression of genes in cultivated soybeans was higher than that in wild soybeans in both pericentromeric regions and chromosomal arms (Supplementary Fig. S5). Furthermore, we found that the number of DEGs in G15 between cultivated and wild soybeans was significantly correlated with gene density on each chromosome (Table 1).

**Table 1.** Correlation of DEG numbers at G15 and gene density in domestication sweeps on chromosomes

Chromosome	Gene number	Expressed genes	DEGs	The correlation of DEG numbers and gene density		Selection sweeps	Genes in sweeps	DEGs in sweeps	The correlation of DEG numbers and gene density in selection sweeps	
				*R*	*P*-value				*R*	*P*-value
Chr1	2457	2241	84	0.265**	3.77E-09	7	194	7	0.146**	0.001
Chr2	3123	2927	109	0.338**	6.73E-13	5	107	2	-0.027	0.58
Chr3	2649	2438	92	0.371**	1.60E-14	5	306	7	0.113*	0.02
Chr4	2574	2387	120	0.343**	2.01E-13	5	67	3	0.038	0.42
Chr5	2491	2321	95	0.368**	7.07E-13	12	323	11	0.002	0.90
Chr6	3258	3052	140	0.390**	1.13E-15	8	349	15	0.062	0.19
Chr7	2743	2575	105	0.269**	5.89E-08	4	338	13	0.001	0.98
Chr8	3679	3519	156	0.259**	1.04E-07	5	208	8	0.004	0.94
Chr9	2865	2655	100	0.320**	9.55E-12	7	149	7	0.105*	0.03
Chr10	2989	2778	113	0.381**	9.15E-17	5	189	4	0.066	0.16
Chr11	2570	2433	126	0.401**	5.03E-13	2	48	2	-0.005	0.93
Chr12	2425	2243	100	0.364**	1.58E-12	2	36	0	-0.011	0.83
Chr13	3730	3193	160	0.258**	6.05E-08	11	355	22	-0.003	0.94
Chr14	2245	1747	86	0.292**	1.22E-09	3	32	2	0.002	0.96
Chr15	2774	2154	106	0.346**	5.94E-14	11	511	18	0.000	0.99
Chr16	2223	1817	89	0.316**	4.32E-09	3	109	6	0.005	0.92
Chr17	2627	2044	86	0.301**	4.41E-09	5	251	12	0.006	0.90
Chr18	3023	2378	102	0.353**	1.66E-16	5	110	6	0.006	0.89
Chr19	2642	2094	80	0.316**	3.62E-11	5	201	7	0.003	0.94
Chr20	2502	2002	89	0.408**	6.70E-18	11	105	2	-0.027	0.58

*Significance at the *P*<0.05 level; **significance at the *P*<0.01 level.

To clarify the possible role of G15 DEGs in soybean domestication, we investigated the relationship between DEGs at G15 and the 121 domestication sweeps previously described (Zhou *et al.*, 2015). We found that 157 DEGs were in the identified domestication sweeps (Table 1; Supplementary Fig. S5). These DEGs are predicted to play multiple roles including regulating cell division and seed development (Supplementary Dataset S13). However, DEGs in the selection sweeps on Chr 1, Chr 3, and Chr 9 were significantly correlated with the gene density in these selection sweeps, while no correlation between the DEG number and gene density in selected regions on the remaining chromosomes was observed (Table 1), indicating that DEGs associated with soybean domestication may be randomly distributed on chromosomes.

### Linking DEGs in domestication sweeps with seed trait-related QTLs

**Fig. 6.**
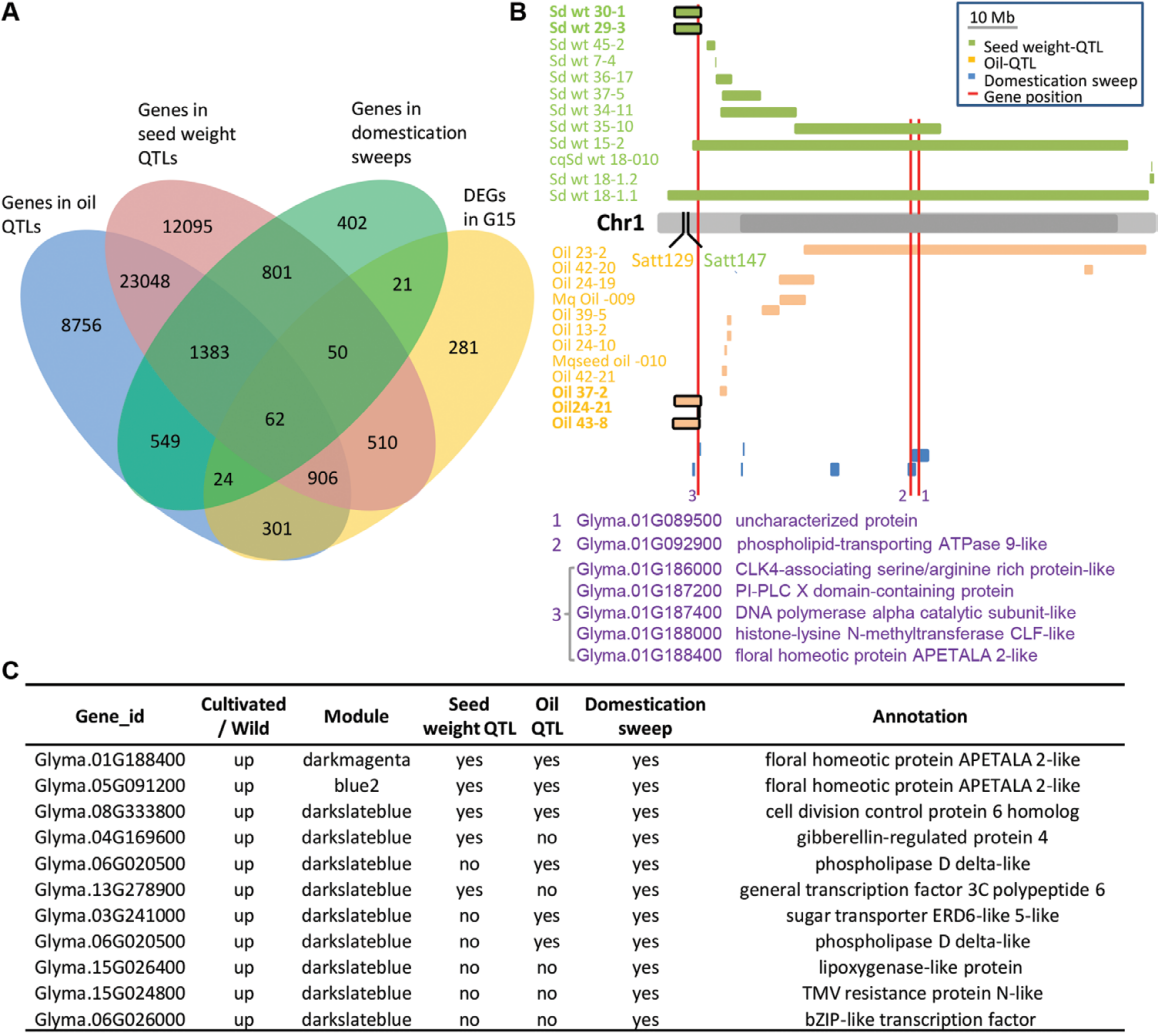
Linkage analyses of DEGs in G15 fruits between *G. soja* and *G. max* with QTLs and domestication sweeps. (A) Venn diagram of QTLs, selection sweeps, genes, and DEGs. Blue indicates genes in oil QTLs, pink indicates genes in seed weight QTLs, green indicates genes in domestication sweeps, and yellow indicates DEGs. The number of genes and DEGs is indicated in each region. (B) Linkage map of DEGs in domestication sweeps and QTLs controlling seed weight and oil content on chromosome 1 (Chr1). Light gray indicates the chromosome and deep gray indicates the pericentromeric regions. Boxes in orange, green, and blue represent oil QTL, seed weight QTL, and domestication sweeps, respectively. The red line represents the DEG position. A black box represents the major QTLs. (C) Selected list of candidate DEGs having putatively important functions during G15 responsible for fruit divergence between *G. soja* and *G. max*. The full DEGs are available in Supplementary Dataset S13.

To understand further the relationship between the observed DEGs and soybean domestication traits such as seed weight and oil content, we performed linkage analysis of DEGs, related QTLs, and selection sweeps. Scrutinizing QTL information in Soybase, we found 292 QTLs controlling seed weight, 314 QTLs controlling oil content, and 121 selection sweeps. Excluding 17 DEGs on scaffolds, 2138 DEGs at G15 between wild and cultivated soybeans were found to be on chromosomes (Fig. 6A; Supplementary Dataset S11). A total of 1528 DEGs were found in seed weight QTL regions (Fig. 6A). Seed weight QTLs without linked DEGs were found on Chr1, Chr2, Chr3, Chr4, Chr7, and Chr8, while DEGs were found in each seed weight QTL region on the remaining 14 chromosomes (Supplementary Fig. S6A). We found that 1293 DEGs were in oil QTLs (Fig. 6A). DEGs were found in each oil QTL region on six chromosomes (Chr3, Chr7, Chr8, Chr9, Chr16, and Chr19), and 1–20 oil QTLs without DGE links were found on the remaining chromosomes (Supplementary Fig. S6B). These observations hinted that different agronomic traits might be differentially associated with distribution patterns of DEGs on chromosomes. However, the selection sweeps without linked DEGs were distributed on all chromosomes (Supplementary Fig. S6C). No DEG was found in the two selection sweeps on Chr12, and around half of the selection sweeps had DEGs (Supplementary Fig. S6C). Moreover, 62 DEGs at G15 were co-located with these QTLs and selection sweeps (Fig. 6A; Supplementary Dataset S13). These analyses further indicate a potential role for the detected DEGs in soybean fruit/seed development and evolution.

To show the relationship between DEGs, QTLs, and selection sweeps visually, we modeled chromosome 1 as representative. Seven DEGs on Chr 1 associated with selection sweeps were overlapped with major QTLs controlling both seed weight (seed weight 29-3 and seed weight 30-1) linked with Satt147 and oil content (seed oil 24-21, seed oil 37-2, and seed oil 43-8) linked with Satt129 (Fig. 6B). The seven DEGs annotated were diverse; however, the gene Glyma.01G188400 encoding AP2-like protein was linked to the major QTL contributing to both seed weight and oil content (Fig. 6B). Its Arabidopsis homolog plays an important role in determining seed size, seed weight, and the accumulation of seed-oil and protein (Jofuku *et al.*, 1994, 2005). Only 82 DEGs between *G. max* and *G. soja* were found at G5, which shared 20 DGEs with G15 Supplementary Fig. S7; Supplementary Dataset S11), but only one (Glyma.13G321100) in a sweep region was co-distributed with one seed weight QTL (Supplementary Dataset S14). This gene putatively encodes an α-farnesene synthase, and no functional involvement has been found with seed development. Therefore, differential expression of genes at G15 may largely account for the divergent fruit morphology between *G. max* and *G. soja*.

The DEGs linked with seed trait-related QTLs and found in selection sweeps are apparently prime target genes to explain the fruit and seed divergence of soybeans. Considering the analyses of DEGs at G15, WGCNA, and linkage with QTLs and selection sweeps, we proposed 157 candidate genes that vary at the transcriptome level that affected the developmental evolution of soybean fruits and seeds (Fig. 6A; Supplementary Dataset S13). Among these candidate genes, DEGs that were functionally annotated to cell division, the accumulation of storage compounds, and hormone response were given priority for analysis of soybean domestication (Fig. 6C). In addition, DEGs at G15 that were not found in selection sweeps but linked with QTLs and seed maturation processes, and were enriched in the divergent gene expression clusters and modules (Supplementary Dataset S11), might be related to soybean improvement. Taken together, the detected DEGs form a genetic resource to help understand soybean domestication and improvement.

## Discussion

Cultivated soybean (*G. max*) was domesticated from its annual wild relative *G. soja* in China (Carter *et al.*, 2004). The underlying genetic variations during soybean domestication have been studied (Sedivy *et al.*, 2017), but are still poorly understood. In the present study, we selected wild soybeans and cultivated soybeans from the northeast of China, a stable and staple soybean production center and where soybeans originated, and compared them at the transcriptome level, shedding new light on soybean domestication and genetic improvement.

### Sequence diversity of transcripts is basically low during soybean evolution

Using the soybean Williams 82 genome as a reference, ~2.5–9.7 million genome-wide SNPs have been found in wild and cultivated soybeans (Lam *et al.*, 2010; Li *et al.*, 2014; Zhou *et al.*, 2015). Our transcriptomic evaluation was around 1/10th of these previous genome-level evaluations; however, it is in line with the fewer SNPs observed in cultivated soybeans compared with wild soybeans (Lam *et al.*, 2010; Li *et al.*, 2014; Zhou *et al.*, 2015). This is believed to have resulted from human selection. However, the general capability of cultivated soybeans to adapt to natural environments has been reduced compared with wild soybeans (Zhao *et al.*, 2015). Signals of adaptive evolution can be detected by an increased ratio of non-synonymous to synonymous substitutions (Bakewell *et al.*, 2007). We found that wild soybeans showed genome-wide accelerated evolution (having a higher dN/dS ratio) relative to cultivated soybeans, suggesting that wild soybeans may have undergone adaptive evolution that allows them to cope with their extremely wide range of conditions and environments. We found 8 and 14 FEGs, respectively, in *G. max* and *G. soja*, but two PSGs were found in each species, and half of the genes were not annotated. The role of these FEGs (including PSGs) in soybean evolution needs investigation, but they are insufficient to explain the divergence of *G. max* and *G. soja* in adaptiveness to natural conditions since they occupy an extremely small portion of soybean genes. The numbers of SNPs and FEGs are basically low among soybeans, though they might have been underestimated in this work due to the close genetic distance between *G. max* and G. *soja* (Li *et al.*, 2014; Zhou *et al.*, 2015), the short transcript sequences, and the low detected amount of non-coding regions that occupy a large fraction of the genome. Nonetheless, ~37% of genes were found to be differentially expressed between wild and cultivated soybeans, indicating that gene expression alteration might have played an essential role in the divergence of *G. max* and *G. soja*. The up-regulated genes in G15 of *G. soja* were enriched in GO terms for stress response, and the adaptive roles of these genes in wild soybean need investigation. However, we found that substantially distinct DEGs were associated with soybean fruit development.

### Distinct gene expression is related to soybean fruit development and morphology

Soybean seed size and related traits are apparently the primary domesticated traits (Lam *et al.*, 2010; Zhou *et al.*, 2015). Organogenesis and morphogenesis occur based on high cell division activity that also contributes to seed size, while the physiological maturity of soybean seeds is the stage of maximal dry weight with accumulated storage compounds. These processes are orchestrated by plant hormones, such as auxin, gibberellin, and ethylene (Slater *et al.*, 2013; Kumar *et al.*, 2014). Accordingly, we found that gene expression patterns were mostly divergent at G5 and G15, especially at G15 (15 d after fertilization). Our results agree with a previous study that revealed that seed size is largely determined at an early developmental stage (at 5–7 d after fertilization and at 10–14 d after fertilization) by comparing two cultivated soybeans (Du *et al.*, 2017). Much evidence suggests that gene expression alteration is essential to drive phenotypic variation during evolution (Cong *et al.*, 2008; Studer *et al.*, 2011; Lin *et al.*, 2012; Koenig *et al.*, 2013). The DEGs in fruit development between wild and cultivated soybeans are presumed to play roles in fruit-related trait divergence in the two closely related species. Species-specifically expressed genes were rarely found. However, we identified the three most divergent gene expression clusters (C1, C2, and C4) in soybeans and 2155 DEGs at G15; a substantial number are associated with cell division, the accumulation of storage compounds, and maturation processes. In particular, in WGCNA, we found that the expression levels of genes in the darkslateblue module at G15 were significantly lower in wild soybeans than in cultivated soybeans, and that genes involved in cell division and hormone (auxin and gibberellin) responses were enriched, such as the previously identified *GmGA20OX* that enhances seed size/weight (Lu *et al.*, 2016). Therefore, these genes, particularly the DEGs in the darkslateblue module and the three divergent gene expression clusters, may have roles in the large fruit/seed size determination of soybeans.

Organ size depends on the number and size of the constituent cells (Mizukami, 2001; Sugimoto-Shirasu and Roberts, 2003). A few DEGs were detected in the most divergent but low expressed cluster (C4). However, the two remaining divergent clusters (C1 and C2) and the major gene co-expression networks, such as the darkslateblue and darkmagenta modules, shared a number of genes related to the cell cycle and cell division, which were differentially expressed between wild and cultivated soybean, such as TFs, CDC genes, and DA1-like; the homologs of these genes have been functionally characterized in Arabidopsis (Blilou *et al.*, 2002; Jofuku *et al.*, 2005; Li *et al.*, 2008; Rojas *et al.*, 2009; Sabelli *et al.*, 2013). Understanding the mechanisms of these genes that govern the cell cycle and their manipulation offers the potential to enhance soybean performance and yield since alteration of cell cycle control has significantly impacted crop evolution and breeding (Hirayama and Shinozaki, 1996; Mizukami, 2001; Sabelli *et al.*, 2013).

Seed size and weight are also highly influenced by the massive accumulation of storage compounds (proteins, oils, and carbohydrates) in cotyledon cells during seed maturation (Dante *et al.*, 2014). We found that seed maturation-related genes such as Glyma.19G147200, Glyma.20G147600, and Glyma.17G155000 were down-regulated in cultivated soybeans, while ethylene-related genes were up-regulated in wild soybeans, correlating with the relatively early and short maturation process in wild soybeans. Gene expression cluster and DEG analyses also generated many important candidate genes, such as sucrose synthase 6-like (Glyma.14G209900), sugar transporter ERD6-like (Glyma.03G241000), and lipoxygenase (Glyma.13G347800), which are related to the synthesis and accumulation of various storage compounds. Extensive investigations of these genes between cultivated and wild soybeans would help us understand how DEGs regulate nutrition accumulation and protein metabolism and their function during seed development and maturation, thus helping us to increase yields in soybeans and related legumes.

The divergent gene expression clusters and the distinctly co-expressed gene networks and associated DEGs that were identified in the present study may form a molecular basis for altering fruit-related traits, morphologically and physiologically. Most of them can be considered for soybean breeding and improvement, while some may have been recruited in soybean domestication.

### Selection of DEGs plays essential roles in soybean domestication

Genomic regional differences in gene expression correlate to gene density in humans and chickens (Caron *et al.*, 2001; Nie *et al.*, 2010), and a significant clustering of the DEGs across the genome has not been found in rice (Guo *et al.*, 2016), suggesting a random distribution of DEGs on chromosomes. However, this is inconsistent with the observation that genomic regions involved in ecological speciation are non-randomly distributed across the genome in whitefish (Derome *et al.*, 2008; Whiteley *et al.*, 2008). The genome-wide association of genetic networks underlying agronomic traits such as seed development and oil content in soybeans suggests that these traits may be genetically co-regulated, and that loci associated with these phenotypes are clustered according to the phylogenetic relationship of traits rather than distributed randomly on chromosomes (Fang *et al.*, 2017). In the present work, transcriptome-wide analysis revealed that the number of DEGs was highly correlated with gene density on chromosomes, providing a first glimpse of the spatiotemporal regulatory landscape of gene expression on chromosomes in cultivated soybeans compared with wild soybeans. However, not all detected DEGs could be causal genes for the divergence of wild and cultivated soybeans. Currently, we are not able to determine the causal genes and to exclude the clustering distribution of these genes on chromosomes. Nonetheless, exploitation of expression quantitative trait locus (eQTL) mapping methods and gene expression analysis in a GWAS (genome-wide association study), namely expression-based GWAS (eGWAS), would be helpful.

A selective sweep is the reduction or elimination of variation among nucleotides near a mutation in DNA and is usually associated with domestication. One hundred and twenty-one selection sweeps have been identified in soybean genomes (Zhou *et al.*, 2015), and we found that none of the fast-evolving genes was in any of these selection sweeps. However, 157 DEGs overall from 2155 DEGs at G15 did fall into the putative regions experiencing domestication sweeps. Moreover, 136 DEGs at G15 in selection sweeps overlapped with QTLs of seed weight and oil content. Limited DEGs at G5 between *G. max* and *G. soja* were linked with QTLs and selective sweeps, and, furthermore, the number of DEGs at G15 had no correlation with the gene density (number) in selection sweep regions on most chromosomes. These observations suggest specific roles for DEGs at G15 in soybean domestication. Our linkage analysis of DEGs overlapping with QTLs and selection sweeps might not be precise, but it offers us many candidate genes to aid in our understanding of the evolution of soybean fruit-related traits. For example, the *AP2* homologous gene on Chr1 might be an important regulator of seed development since it is linked with major QTLs controlling seed weight and oil content (H. Kim *et al.*, 2010; Liu *et al.*, 2011; Qi *et al.*, 2011; Eskandari *et al.*, 2013; Mao *et al.*, 2013) and because its Arabidopsis homolog controls seed size and development (Jofuku *et al.*, 1994, 2005).

The evolutionary and developmental roles in soybean domestication of the genetic variations identified here need further investigation. Variation in either *cis*-regulatory elements or *trans*-acting factors could lead to differential expression of genes. However, in contrast to the extremely limited sequence divergence at coding regions (Kim *et al.*, 2012; Li *et al.*, 2014; this study), we found that nearly 40% of genes were differentially expressed between wild and cultivated soybeans, and ~7.3% of the DEGs during fruit and seed development were linked with selection sweeps, which correlated with the fruit-related trait divergence between *G. soja* and *G. max*. We thus conclude that gene expression changes largely due to *cis*-element divergence are the key drivers of phenotypic variation during soybean domestication.

### Conclusions

Transcriptomic comparisons between wild and cultivated soybeans uncover both sequence and gene expression variations involved in the domestication of soybeans. In this work, we found that gene expression variation is the primary evolutionary event during the divergence of wild and cultivated soybeans in contrast to sequence diversity in the coding regions. Distinct gene expression divergence was detected around the cotyledon stage of seed development (~15 d after fertilization) between wild and cultivated soybeans, and the cell cycle network in particular was found to be differentially expressed, which is related to fruit (seed) size variations. Thus, gene expression changes were essential drivers of the physiological and morphological variations related to fruit and seed during soybean domestication. A set of candidate genes for soybean seed domestication are putatively proposed based on our multiple analyses, providing genetic resources for soybean improvement and breeding. Increasing the sample size, and using either eQTL or eGWAS will be more effective to find key candidate genes for soybean domestication and to improve soybean agronomic traits. Functional verification of the genome- and transcriptome-wide detected variations will be the main goal of future investigations.

## Supplementary data

Supplementary data are available at *JXB* online..

Fig. S1. Flowchart of this work and key steps for data processing.

Fig. S2. Polymorphisms between *G. max* and *G. soja*.

Fig. S3. Pre-analyses for WCNGA.

Fig. S4. Distribution of DEGs in gene expression clusters and the co-expression modules.

Fig. S5. Global depiction of gene expression at G15 in soybeans.

Fig. S6. Distribution of DEGs at G15 between wild and cultivated soybeans on chromosomes.

Fig. S7. QTLs, selection sweeps, genes, and DEGs in G5 fruits between *G. soja* and *G. max*.

Dataset S1. Detailed information of soybeans used in the present work.

Dataset S2. Number of reads sequenced and mapped on the Williams 82 genome of RNA-seq data.

Dataset S3. Genes detected in soybeans.

Dataset S4. SNP calling in 40 RNA-seq samples.

Dataset S5. dN/dS evaluation of single-copy orthologous genes in soybeans.

Dataset S6. Annotation of genes in the 12 gene expression clusters.

Dataset S7. GO enriched terms of genes in the 12 gene expression clusters.

Dataset S8. Genes in the 23 modules.

Dataset S9. Hub genes in the 23 modules.

Dataset S10. GO analysis of the darkslateblue module.

Dataset S11. Detected DEGs at G15, G5, H, WH, and JJ between cultivated and wild soybeans.

Dataset S12. Enriched GO terms of the identified DEGs at G15 in soybeans.

Dataset S13. DEGs at G15 in domestication sweeps and QTLs controlling seed weight and oil content.

Dataset S14. DEGs at G5 in domestication sweeps and QTLs controlling seed weight and oil content.

Supplementary Figures

Supplementary Data S1

Supplementary Data S2

Supplementary Data S3

Supplementary Data S4

Supplementary Data S5

Supplementary Data S6

Supplementary Data S7

Supplementary Data S8

Supplementary Data S9

Supplementary Data S10

Supplementary Data S11

Supplementary Data S12

Supplementary Data S13

Supplementary Data S14

## Abbreviations

DEG: differentially expressed genes
GO: gene ontology
QTL: quantitative trait locus
SNP: single nucleotide polymorphism
TF: transcription factor
