# Supplementary material for: Transcriptomic comparison reveals genetic variation potentially underlying seed developmental evolution of soybeans
Source: J Exp Bot. 2018 Aug 3;69(21):5089–104. doi: 10.1093/jxb/ery291 (PMC6184420; doi:10.1093/jxb/ery291)
Supplement: Supplementary Figures [file ery291_suppl_figures.pdf]

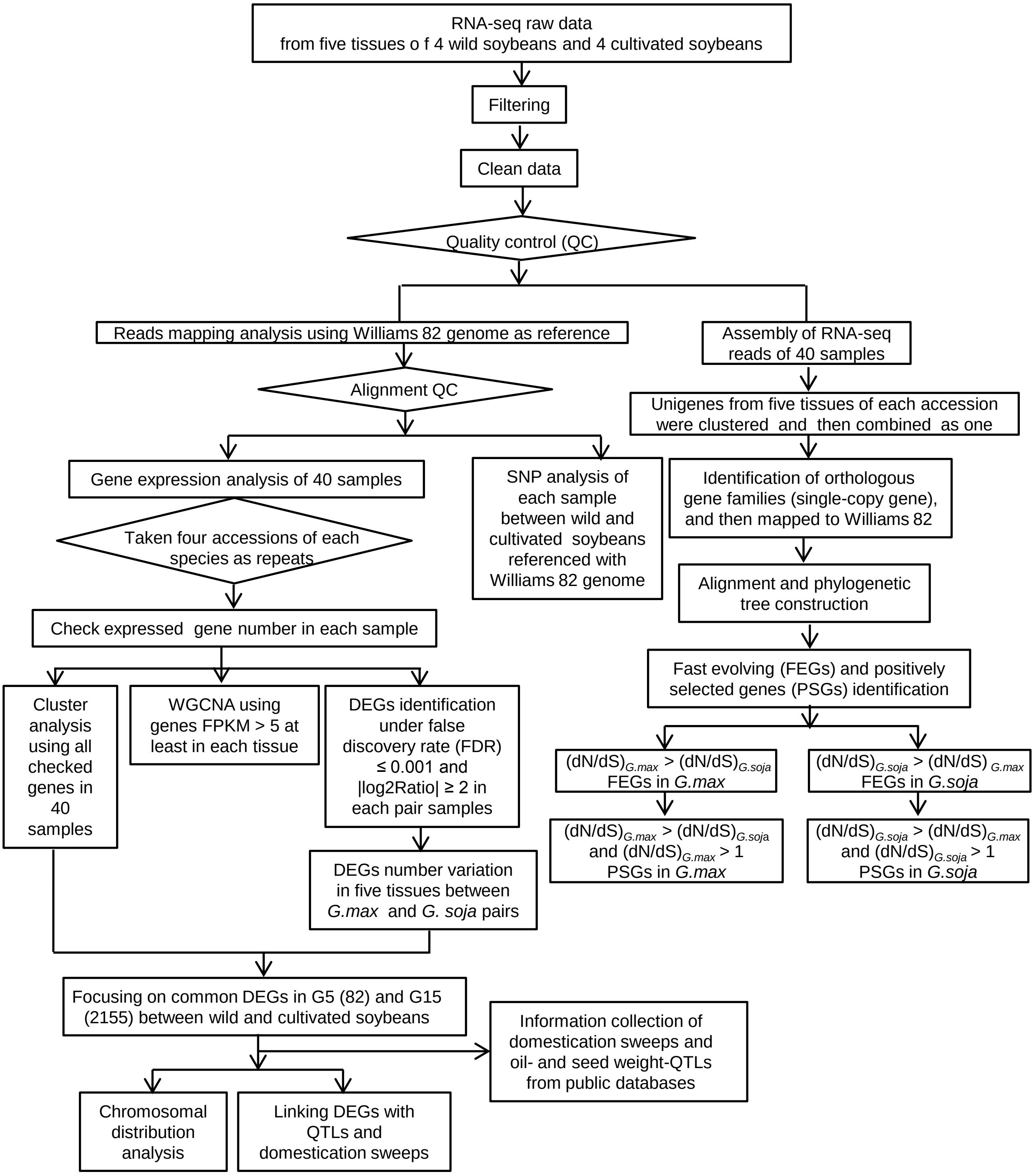

Figure S1 Flowchart of this work and key steps for data processing.
The experimental procedures are described in the Materials and Methods. SNP, single nucleotide polymorphism; DEGs, differentially expressed genes; QTL, quantitative trait loci; G5, fruits after 5-days fertilization; G15, fruits after 15-days fertilization; WGCNA, weighted gene coexpression network analysis.

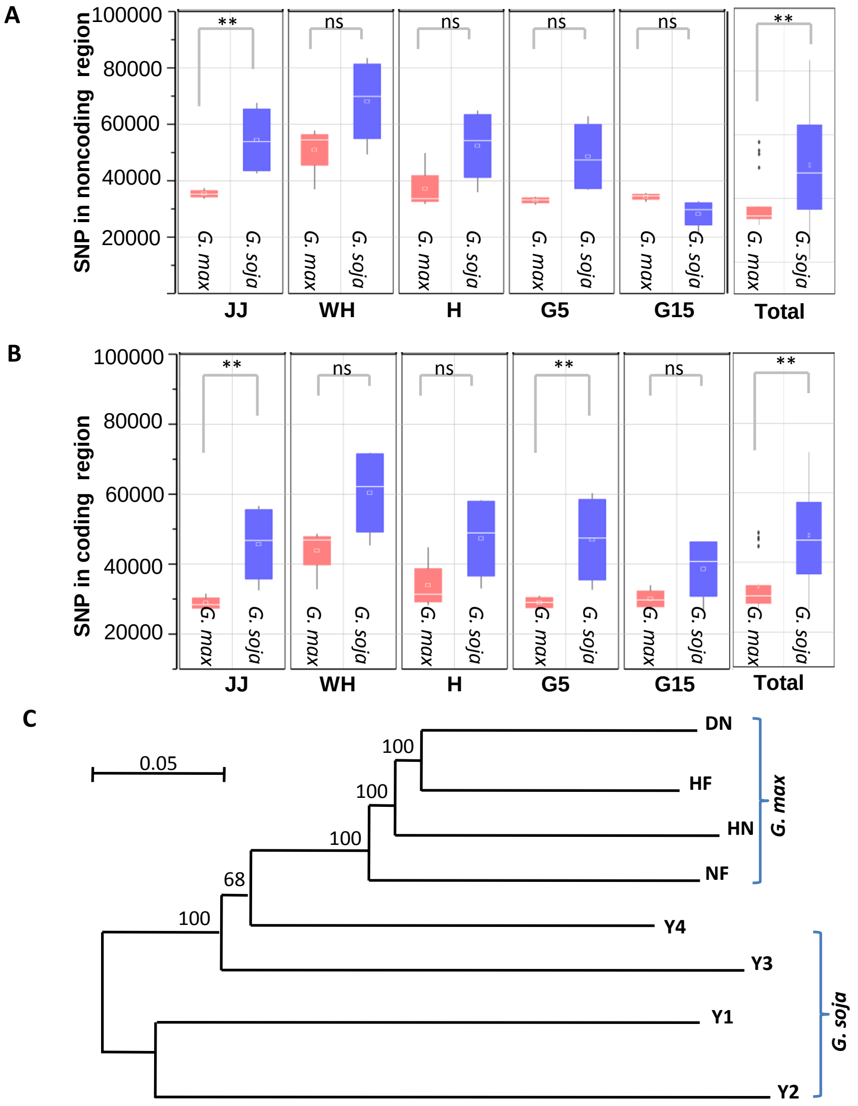

Figure S2 Polymorphisms between *G. max* and *G. soja*.
(A) Boxplot of the number of SNPs found in non-coding regions. (**B**) Boxplot of the number of SNPs found coding regions. ** represents significant difference at $P < 0.01$; ns, no significant difference. **(C)** Neighbor-joining (NJ) tree of wild and cultivated soybeans using transcriptomic data. The scale bar represents the expected number of substitutions per site.

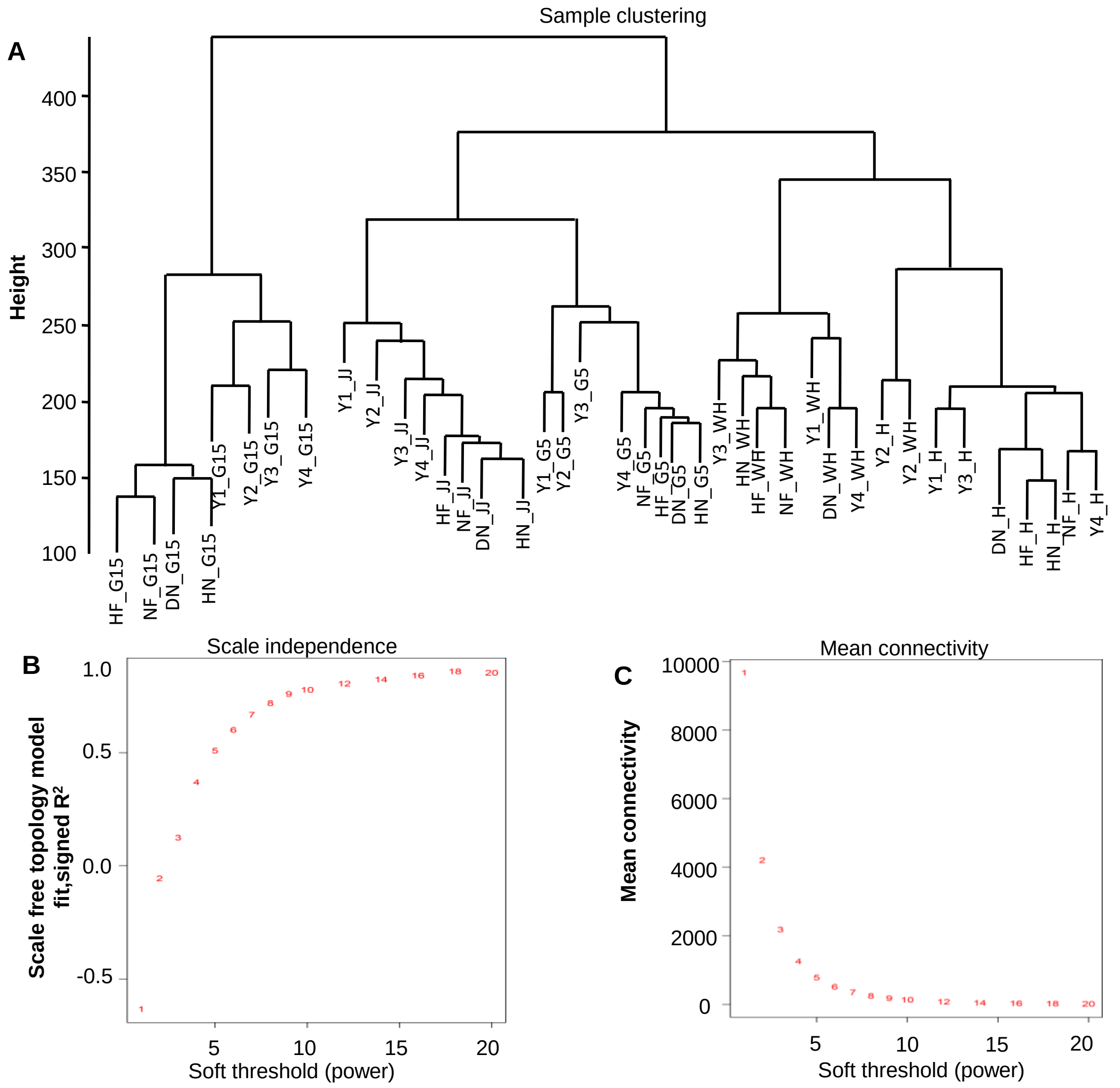

Figure S3 Pre-analyses for WCNGA.
(**A**) Hierarchical clustering of samples. (**B, C**) Selection of the soft-thresholding powers. (**B**) The scale-free fit index vs. soft-thresholding power. (**C**) The mean connectivity vs. soft-thresholding power.

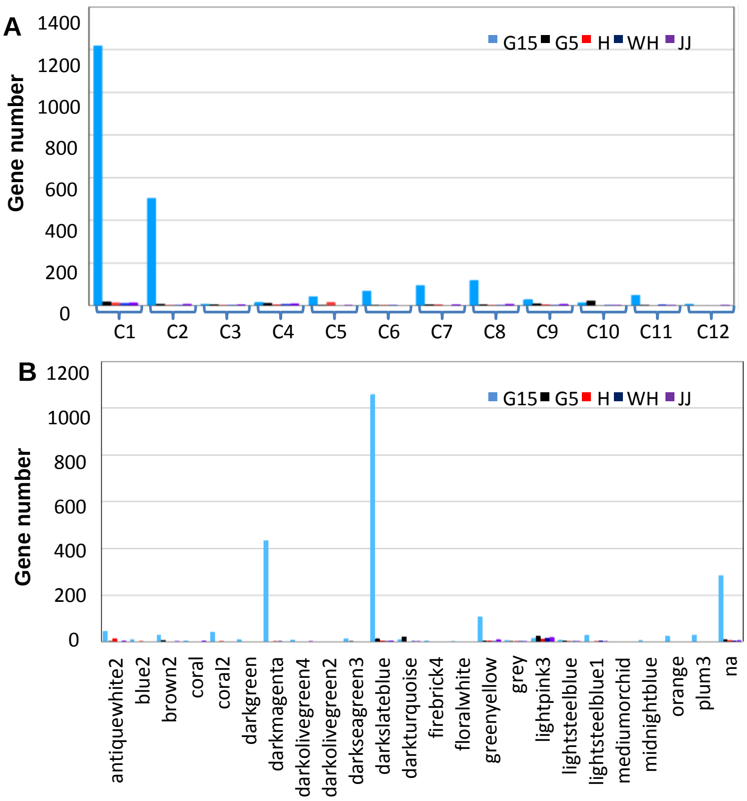

C

Module	Hub genes	DEGs
antiquewhite2	27	0
blue2	9	1
brown2	17	0
coral	8	1
coral2	4	0
darkgreen	34	0
darkmagenta	42	24
darkolivegreen2	30	0
darkolivegreen4	2	0
darkseagreen3	4	0
darkslateblue	18	12
darkturquoise	11	0
firebrick4	3	0
greenyellow	23	0
lightpink3	2	2
lightsteelblue	10	2
lightsteelblue1	7	2
mediumorchid	2	0
midnightblue	32	0
orange	2	1
Total	287	45

Figure S4 Distribution of DEGs in gene expression clusters and the coexpression modules. **(A)** Histogram of DEG number in 12 clusters. **(B)** Histogram of DEG number in 23 modules. (**C**) DEG number in the hub genes of each module.

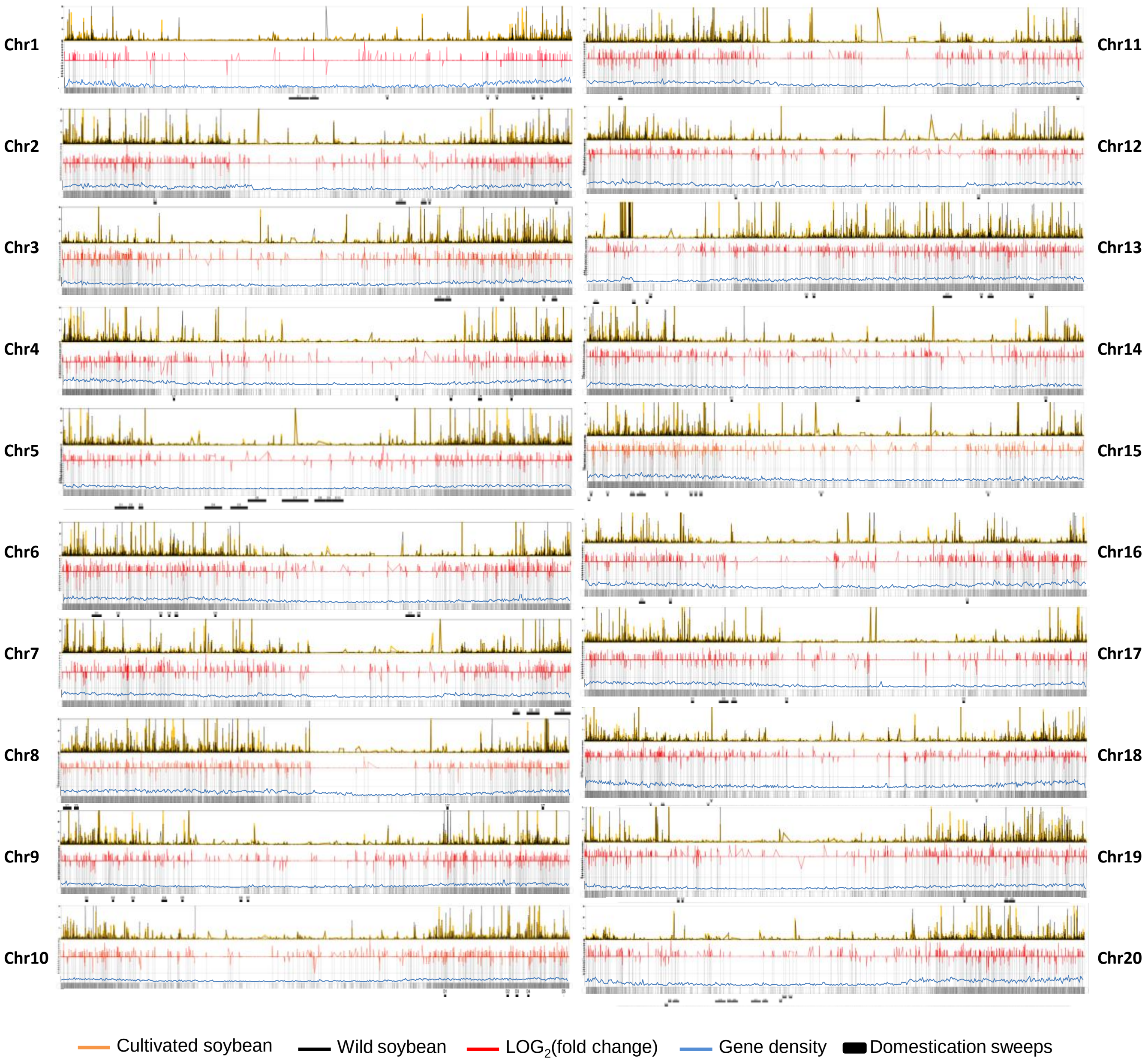

Figure S5 Global depiction of gene expression at G15 in soybeans.
Each of the 20 soybean chromosomes was scanned by 1-Mb sliding windows with 0.1 Mb steps. Average FPKM of the expressed genes is shown in cultivated soybeans (orange) and wild soybeans (black). DEGs and the log_2 (fold change) are indicated in red and gene density is in blue. Black orthogons represent domestication sweeps. Gene density per 1-Mb window is shown on the primary Y-axis, and gene count is displayed on the secondary Y-axis. The X-axis represents the chromosome length in Mb.

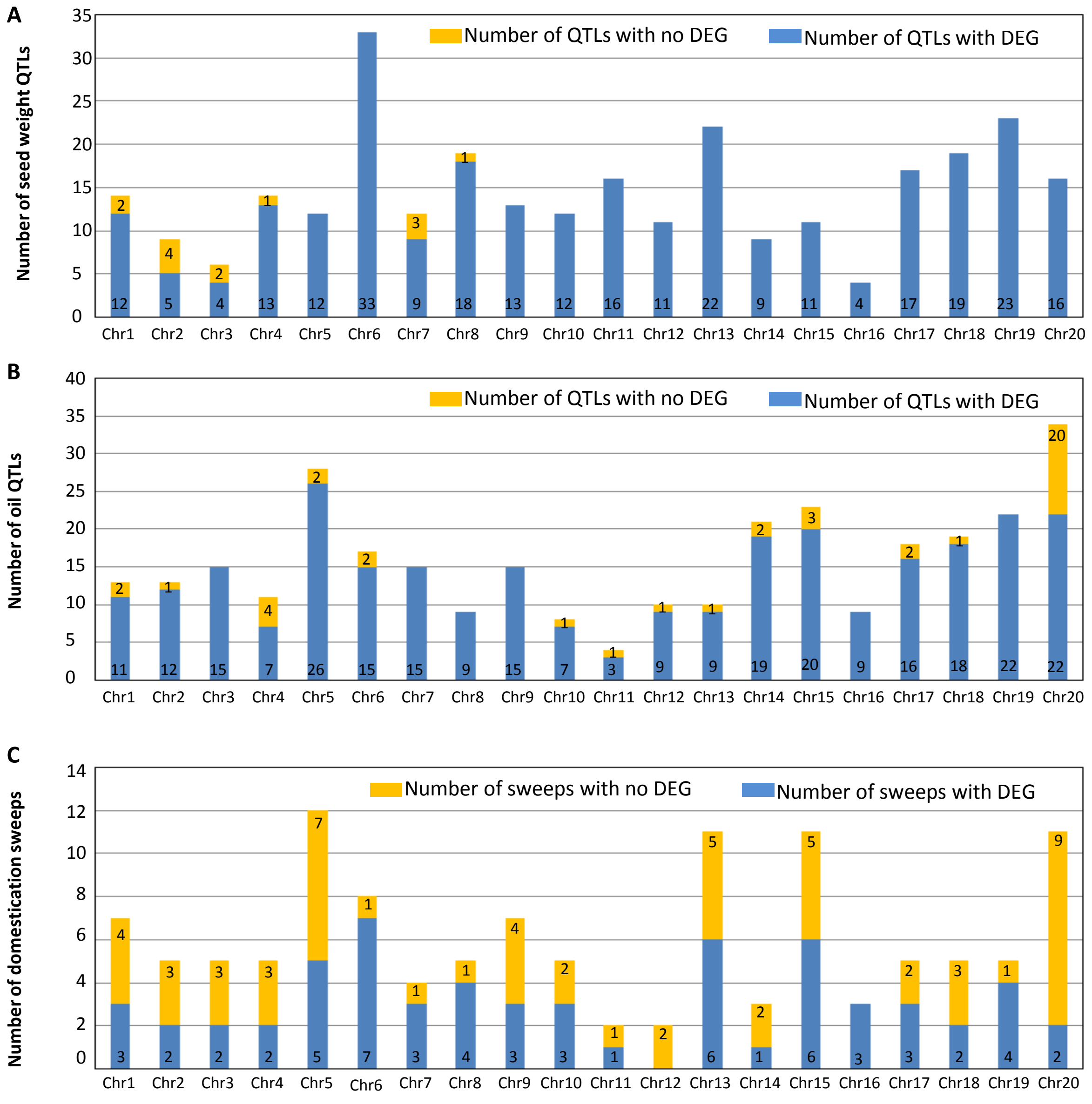

Figure S6 Distribution of DEGs at G15 between wild and cultivated soybeans on chromosomes. (**A**) DEG and seed weight QTLs. (**B**) DEG and oil QTLs. (**C**) DEG and selection sweeps. Orange represents QTLs or sweeps without DEGs. Blue represents QTLs or sweeps having DEGs. Triangle indicates total number of QTL or sweeps on chromosomes (chr1-chr20).

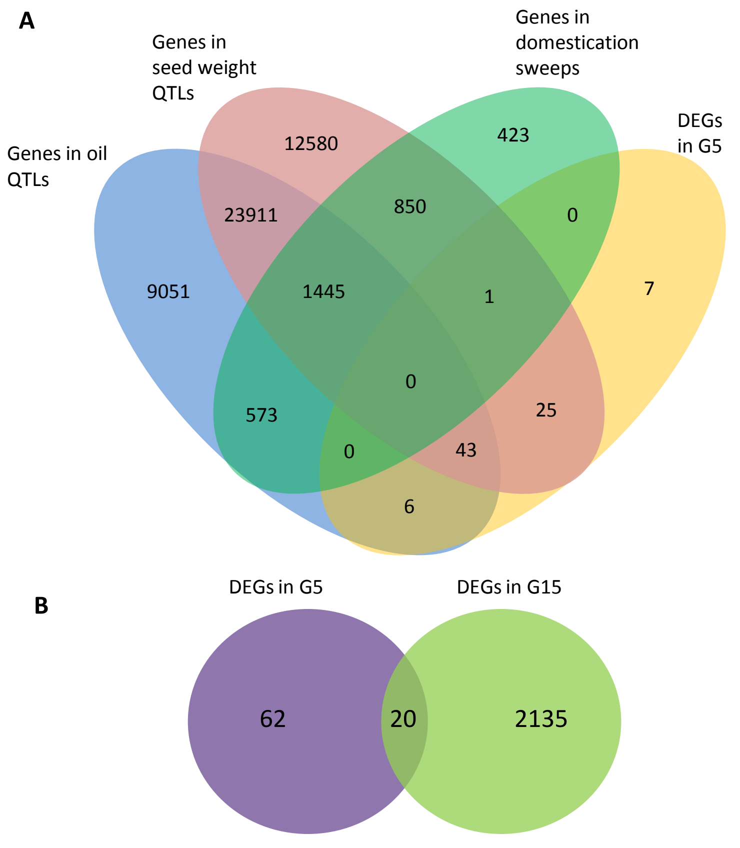

Figure S7 QTLs, selection sweeps, genes, and DEGs in G5 fruits between *G. soja* and *G. max*. (**A**) Venn diagram QTLs, selection sweeps, genes, and DEGs. Blue means genes in oil QTLs, pink means genes in seed weight QTLs, green means genes in domestication sweeps, and yellow means DEGs. (**B**) Relationship of DEGs between G5 and G15. The number of genes and DEGs is indicated in each region.
